# Supplementary material for: Aneurysmal Subarachnoid Hemorrhage in Hospitalized Patients on Anticoagulants—A Two Center Matched Case-Control Study
Source: J Clin Med. 2023 Feb 13;12(4):1476. doi: 10.3390/jcm12041476 (PMC9958876; doi:10.3390/jcm12041476)
Supplement: Supplementary file 1 [file jcm-12-01476-s001.zip › jcm-2177595-supplementary.pdf]

Supplemental Table S1.

	OR	95% CI	p-value
NOAC use	0.633	0.135 to 2.961	0.562
DCI occurrence	5.333	0.343 to 82.827	0.232*
Mechanical ventilation	2.057	0.575 to 7.364	0.268*
Sepsis	0.333	0.028 to 3.990	0.386*
WFNS grading (dichotomous)	7.500	1.715 to 32.796	0.007
	**OR**	**95% CI**	**p-value**
VKA	3.067	0.869 to 10.823	0.081
DCI occurrence	1.280	0.457 to 3.585	0.639*
Mechanical ventilation	12.042	3.079 to 47.088	< 0.001
Sepsis	1.133	0.277 to 4.629	0.862*
WFNS grading (dichotomous)	9.200	2.882 to 29.364	< 0.001

Supplemental Table S1. Results of univariate logistic regression assessing the effect of direct oral anticoagulant (DOAC) or vitamin K antagonist (VKA) treatment on the occurrence of unfavorable outcome (GOS_{1-3}) after 12 months.

CI, confidence interval; DCI, delayed cerebral ischemia; DOAC, direct oral anticoagulants; GOS, Glasgow outcome scale, OR, odds ratio; VKA, vitamin K antagonists; WFNS, world federation of neurosurgical societies.
* These explanatory variables were still included into the multivariate logistic regression model based on their clinical relevance and anticipated effect on outcome.

Supplemental Table S2.

	OR	95% CI	p-value
DOAC use	2.696	0.300 to 24.228	0.376
DCI occurrence	8.290	0.940 to 73.122	0.057
Mechanical ventilation	2.497	0.414 to 15.053	0.318
Sepsis	8.287	0.194 to 354.160	0.270
WFNS grading (dichotomous)	11.047	1.520 to 80.286	0.018
	**OR**	**95% CI**	**p-value**
VKA use	2.780	0.631 to 12.234	0.176
DCI occurrence	1.016	0.285 to 3.643	0.980
Mechanical ventilation	6.769	1.483 to 30.892	0.014
Sepsis	1.207	0.205 to 7.104	0.043
WFNS grading (dichotomous)	4.833	1.376 to 16.977	0.014

Supplemental Table S2. Results of multivariable logistic regression assessing the effect of direct oral anticoagulant (DOAC) or vitamin K antagonist (VKA) treatment on the occurrence of unfavorable outcome (GOS_{1-3}) after 12 months.

CI, confidence interval; DCI, delayed cerebral ischemia; DOAC, direct oral anticoagulants; GOS, Glasgow outcome scale, OR, odds ratio; VKA, vitamin K antagonists; WFNS, world federation of neurosurgical societies.
